# Effect of Gamma Knife Radiosurgery and Programmed Cell Death 1 Receptor Antagonists on Metastatic Melanoma

**DOI:** 10.7759/cureus.1943

**Published:** 2017-12-13

**Authors:** Nathan Nordmann, Molly Hubbard, Tyler Nordmann, Paul W Sperduto, H. Brent Clark, Matthew A Hunt

**Affiliations:** 1 Neurosurgery, University of Minnesota Medical School; 2 Department of Neurosurgery, University of Minnesota; 3 Electrical Engineering, University of Minnesota; 4 Minneapolis Radiation Oncology & Gamma Knife Center, University of Minnesota; 5 Pathology, University of Minnesota

**Keywords:** melanoma, radiosurgery, immunotherapy

## Abstract

Learning objectives

To evaluate radiation-induced changes in patients with brain metastasis secondary to malignant melanoma who received treatment with Gamma Knife radiosurgery (GKRS) and programmed cell death 1 (PD-1) receptor antagonists.

Introduction

Stereotactic radiosurgery and chemotherapeutics are used together for treatment of metastatic melanoma and have been linked to delayed radiation-induced vasculitic leukoencephalopathy (DRIVL). There have been reports of more intense interactions with new immunotherapeutics targeting PD-1 receptors, but their interactions have not been well described and may result in an accelerated response to GKRS. Here we present data on subjects treated with this combination from a single institution.

Methods

Records from patients who underwent treatment for metastatic melanoma to the brain with GKRS from 2011 to 2016 were reviewed. Demographics, date of brain metastasis diagnosis, cause of death when applicable, immunotherapeutics, and imaging findings were recorded. The timing of radiation therapy and medications were also documented.

Results

A total of 79 subjects were treated with GKRS, and 66 underwent treatment with both GKRS and immunotherapy. Regarding the 30 patients treated with anti-PD-1 immunotherapy, 21 patients received pembrolizumab, seven patients received nivolumab, and two patients received pembrolizumab and nivolumab. Serial imaging was available for interpretation in 25 patients, with 13 subjects who received GKRS and anti-PD-1 immunotherapy less than six weeks of each other. While four subjects had indeterminate/mixed findings on subsequent magnetic resonance imaging (MRI), nine subjects were noted to have progression. Two of these patients showed progression but subsequent imaging revealed a decrease in progression or improvement on MRI to previously targeted lesions by GKRS. None of the 13 subjects had surgery following their combined therapies.

Conclusions

This data suggests that there is need for further investigation of the role for concurrent treatment with PD-1 inhibitors and GKRS to enhance the treatment of metastatic melanoma. We present data on 13 patients who appear to have some radiologic benefit to this treatment combination, two of whom had radiographic pseudoprogression.

## Introduction

Metastatic spread of tumors to the brain presents a treatment challenge, as intracranial spread may often be the only location of metastatic disease. Certain tumor types are responsive to radiation or chemotherapeutic agents, but the blood brain barrier prevents adequate penetration of chemotherapeutic agents. Melanoma is particularly difficult to treat, as it is historically not well responsive to fractionated radiation and older chemotherapeutic medications. Intracranial lesions are identified in up to 75% of melanoma patients in clinical trials [1] and contribute to death in 94% of subjects with metastases [2-4]. With intentions to prolong patient survival and improve quality of life, immune-modulating therapies are being added to systemic treatment regimens and are becoming the standard of care for patients with known brain metastases. One subclass known as programmed cell death 1 (PD-1) inhibitors is gaining attention not only for a durable response and high response rate in patients with brain metastases but also its ability to create a clinical effect and transient radiographic enhancement when combined with Gamma Knife radiosurgery (GKRS) [5].

In general, radiation necrosis is typically defined as necrotic changes that occur in tumor cells and perilesional brain tissue from the cytotoxic effects of radiation. This is an irreversible process, commonly reported to manifest months to years after treatment with radiation and chemotherapy [6]. It is seen after treatment for glioblastoma as well as metastatic disease [7-8]. Upon histologic examination, vascular abnormalities, marked astrocytosis, hyalinization and sclerosis of blood vessels, and demyelination of axons are all findings that may precede the death of tissue caused by radiation therapy [5, 9]. While each of these changes may be distinct on a molecular level, they can manifest as changes on magnetic resonance imaging (MRI) similar to the MRI findings of biologically active tumor cells. Accordingly, this radiographic mimicry may preemptively warrant a biopsy, only to find that the pathology is consistent with a delayed radiation-induced vasculitic leukoencephalopathy (DRIVL) from GKRS and no evidence of recurrent or viable tumor [10-12].

Similar findings of false progression have been noted to occur with the previously mentioned PD-1 inhibitors, but the underlying mechanism likely is different. Pembrolizumab (KEYTRUDA, Merck & Co., Inc.) and nivolumab (OPDIVO, Bristol-Myers Squibb Company) are monoclonal antibodies that target the co-inhibitory pathway that uses the programmed cell death 1 receptor and are now being used for treatment of metastatic melanoma. These antibodies block inhibition of cytotoxic T lymphocytes (CTL) and result in a robust immune response [13]. These drugs have been reported to show an initial increase in size of the radiographic lesion and surrounding enhancement followed by stabilization or resolution over time without any further intervention. These findings are consistent with pseudoprogression, a term that denotes a transient progression on imaging that stabilizes or resolves over time.

Irrespective of the underlying mechanism that leads to this transient progression on serial imaging, similar findings can be seen after monotherapy with either GKRS or PD-1 antagonists. Recently, there have been reports of more intense radiologic changes on imaging following administration of both GKRS and a PD-1 inhibitor [5]. The interaction has not been well-described pathologically but suggests an accelerated response to GKRS. Whether or not these findings illustrate pseudoprogression from PD-1 immunotherapy or an accelerated radiation-induced necrosis from combined therapy is not trivial, as mistakenly assuming post-treatment changes for disease progression can lead to unnecessary medical intervention and subsequent harm to patients. Here, we present our experience with treatment of metastatic melanoma with GKRS and PD-1 inhibitors at a single institution.

## Materials and methods

This research received institutional review board (IRB) approval (study number 1612M03021) prior to review of medical records. Information was obtained from existing electronic medical records (EMR) of patients who underwent gamma knife radiosurgery (GKRS) from 2011-2016. Information regarding each subject’s age at time of diagnosis and each treatment, gender, date of brain metastasis diagnosis, cause of death (when applicable), immunotherapies, chemotherapies, gamma knife radiosurgery treatment, date and number/location of targeted lesions, craniotomy treatment date and location (when applicable), and imaging findings from neuroradiology interpretations (MRI, or computed tomography (CT) when MRI contraindicated) were recorded. The timing of both immunotherapies and chemotherapies also was documented. If the patient did require surgical intervention, pathological specimens were recorded and reviewed with a neuropathologist as well.

In order to satisfy inclusion criteria, subjects must have been diagnosed with metastatic melanoma to the brain and received treatment with GKRS and a PD-1 antagonist. To assess the effects of this combined treatment regimen, GKRS and PD-1 immunotherapy were allowed a maximum separation of up to, but not including, six weeks. Serial MR and/or CT imaging was then reviewed for subjects meeting these restrictions, one month, three months, six months, nine months, and twelve months status post GKRS. Imaging between or within these timeframes also was taken into account if the radiologist noted a separate finding not mentioned elsewhere. Subjects who received more than one GKRS, repeat GKRS to the same lesion(s), or surgical intervention were not excluded if they met the above criteria. Interval changes regarding tumor size and surrounding edema or hyperintensities were documented on axial T1+contrast and T2 fluid attenuated inversion recovery (FLAIR), respectively.

For this study, pseudoprogression was defined as any transient progression in tumor size and/or perilesional edema after receiving both GKRS and PD-1 immunotherapy. Basic descriptive methods were used in this study to evaluate trends and correlations between patient treatments and outcome, with subjects being placed into three distinct categories: progression, demonstrated by interval increase in lesion size and/or surrounding edema; indeterminate/mixed, demonstrated by limited data for which progression could not be adequately measured or quantified; and imaging unavailable, demonstrated by no data for which progression could be adequately measured or quantified. No progression, demonstrated by either a decrease in size/enhancement of lesion (without any previous or ensuing progression) or complete resolution of lesion, was originally included for categorization but no patient met these standards upon further evaluation.

## Results

A total of 79 subjects (50 male, 29 female) were evaluated with diagnosis of melanoma with brain metastasis. The average age at which patients received the diagnosis of metastatic disease to the brain was 58 years old (standard deviation of 11.68). The youngest and oldest subjects were 27 and 88 years old, respectively. There were 54 subjects (68.4%) who were alive during data collection, while 25 subjects (31.6%) have succumbed to either malignant melanoma or other reasons not documented. Most subjects reported themselves to be White (75; 94.9%), while the race of the remaining subjects (four; 5.1%) was unknown or not reported.

**Figure 1:**
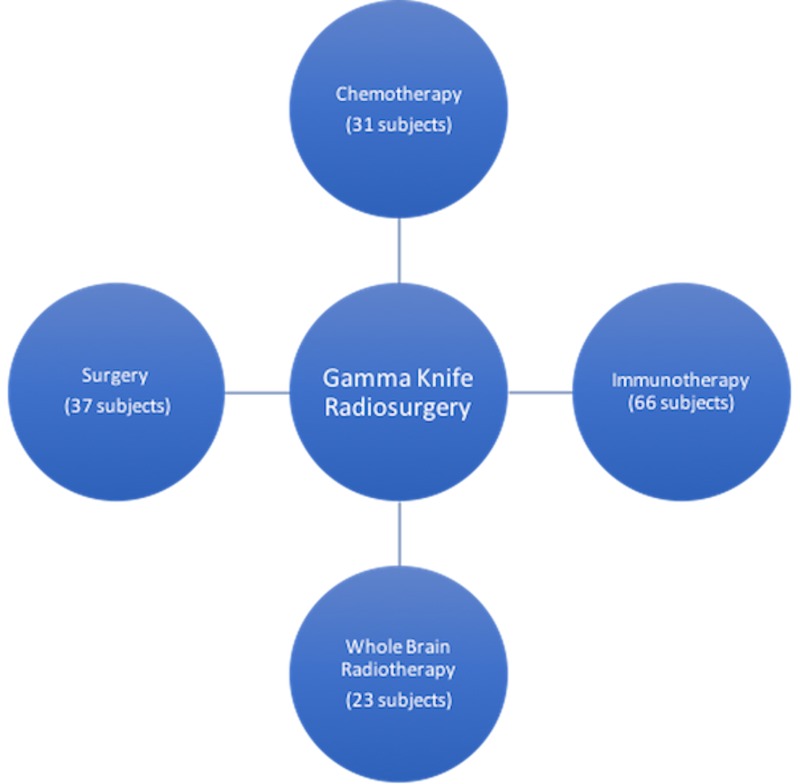
All treatment interventions used for 79 subjects diagnosed with metastatic melanoma.

All 79 patients were diagnosed with biopsy-proven melanoma before receiving GKRS to the targeted brain lesion(s). As illustrated in Figure 1, many patients received immunotherapy, chemotherapy, whole brain radiotherapy (WBRT), and/or surgery in addition to GKRS.

**Figure 2:**
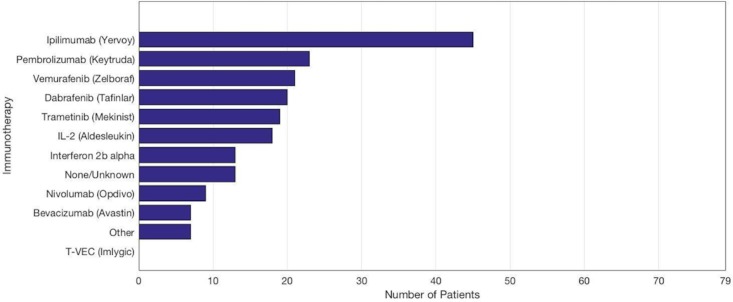
Immunotherapeutics used for the treatment of 79 subjects diagnosed with metastatic melanoma.

In 66 of 79 patients, some form of immunotherapy was given concurrently with GKRS. As illustrated in Figure 2, ipilimumab (Yervoy) was the most common medication used (45 subjects; 57.0%). The category of “other” included less commonly used agents: cobimetinib (two subjects), everolimus (two subjects), ibrutinib (one subject), sorafenib (one subject), and interferon alpha-2a IJ (one subject). Talimogene laherparepvec (T-VEC, Imlygic) was initially included as an immunotherapeutic agent to screen for but no patients were taking this medication upon chart review.

Of importance to this study, 30 patients (38.0%) underwent treatment with a PD-1 antagonist and GKRS at some point in their medical management: 21 patients received only pembrolizumab, seven patients received only nivolumab, and two patients received pembrolizumab and nivolumab.

For the 30 patients who received PD-1 antagonist immunotherapy and GKRS, the subjects were stratified into groups depending on the window period between administration of both treatment modalities: 18 subjects received both treatments less than six weeks of each other, five subjects were given both within six to twelve weeks, and another seven subjects had GKRS and PD-1 antagonist separated by greater than twelve weeks. Of note, the two subjects who received both pembrolizumab and nivolumab (in addition to GKRS) were placed into the less-than-six-weeks category, as pembrolizumab was administered within one month from completion of GKRS for both patients. According to Alormari, et al., early signs of radiation-induced changes were noted in a subject with cerebral metastatic melanoma who received GKRS and PD-1 immunotherapy separated by less than four weeks [5]. With the intention to increase subjects who met inclusion criteria for this study, the time window between administration of therapies was increased to less than six weeks.

**Figure 3:**
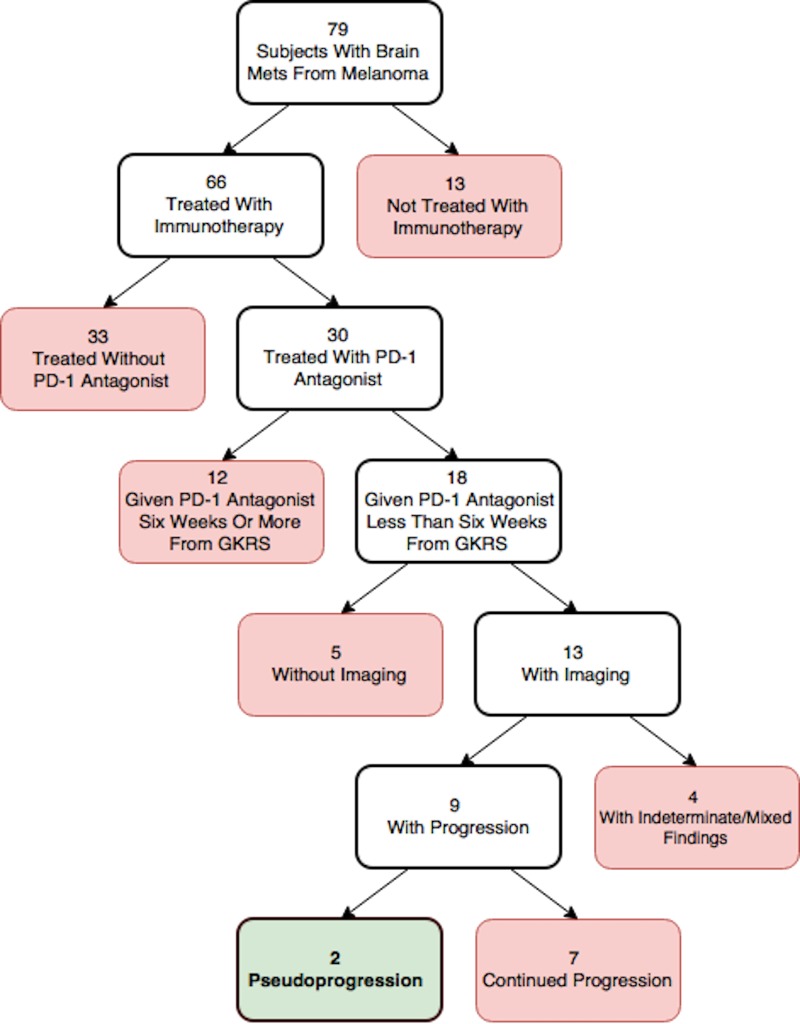
Flowchart illustrating the methodology and subsequent findings for subjects who received Gamma Knife radiosurgery (GKRS) and programmed cell death 1 (PD-1) antagonist less than six weeks of each other.

Of the 18 patients who underwent PD-1 antagonist immunotherapy and GKRS less than six weeks of each other, five subjects did not have imaging available after administration of both treatment modalities. Consequently, a total of 13 subjects were evaluated with imaging for this specific window. Serial imaging revealed four subjects (30.8%) with indeterminate/mixed findings and nine subjects (69.2%) were noted to have progression. Progression was defined as an increase in tumor size and/or surrounding edema without evidence of regression after being subjected to both treatments. The methodology leading to these findings is illustrated in Figure 3.

Specific attention was given to the latter, as two subjects (22.2%) initially showed apparent progression, but subsequent imaging revealed a decrease in progression or improvement in surrounding vasogenic edema to lesions previously targeted by GKRS. Although these changes on imaging favor pseudoprogression from anti-PD1 immunotherapy and/or early signs of radiation-induced necrosis from GKRS, pathologic specimens were not obtained afterward for confirmation. Below is a summary of these two subjects.

Case presentations - pseudoprogression

Case 1

**Figure 4:**
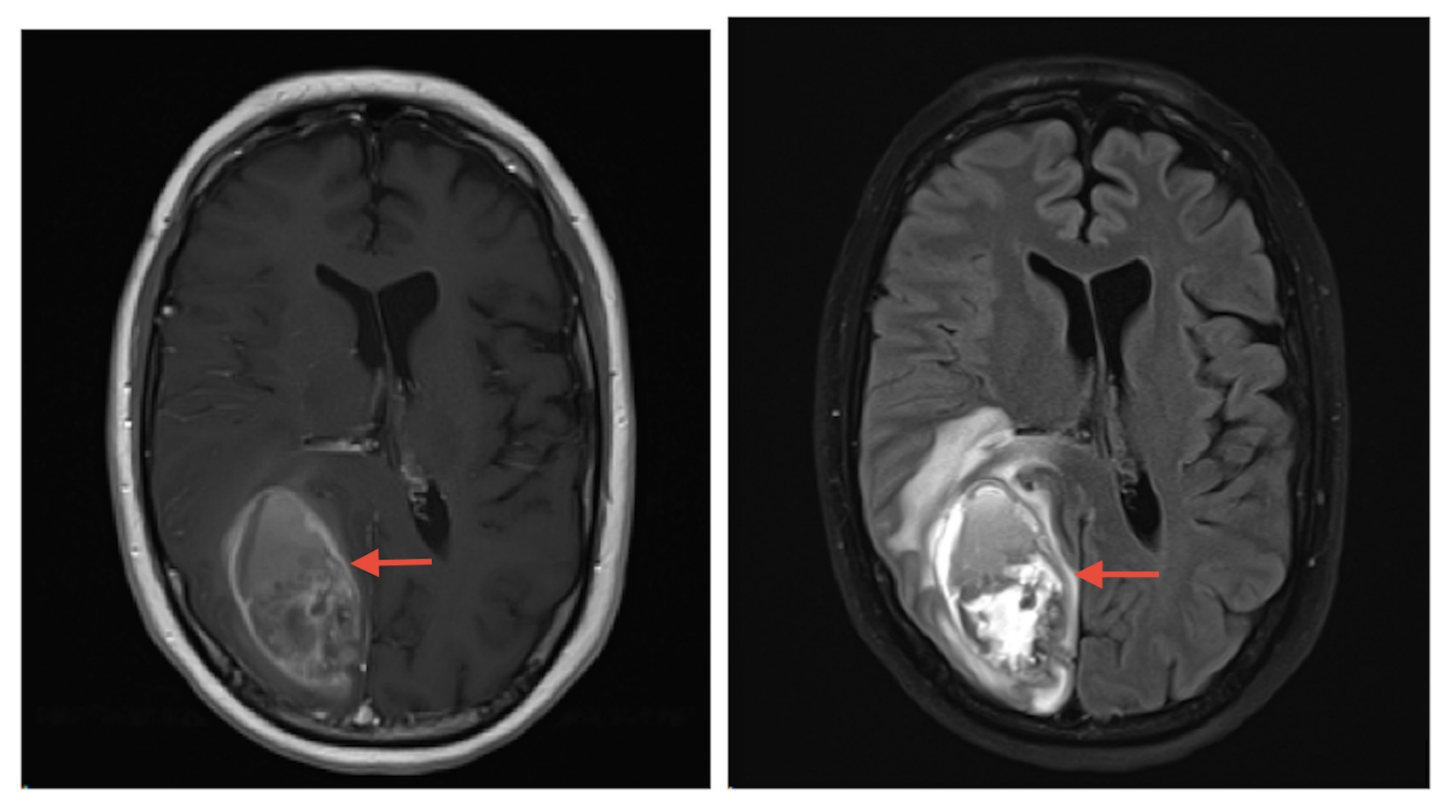
Preoperative magnetic resonance imaging demonstrating post-contrast T1-weighted (left) and T2 fluid attenuated inversion recovery (right) images of 6.3 x 3.5 x 5.3 centimeter hemorrhagic lesion in the right occipital lobe.

**Figure 5:**
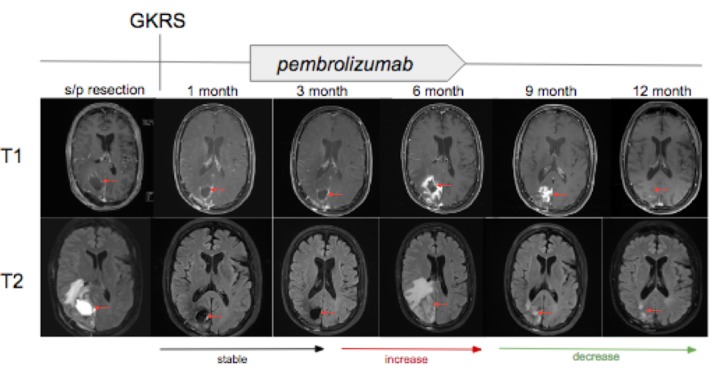
T1-weighted with gadolinium (top) and T2 fluid attenuated inversion recovery images (bottom) associated with the right occipital lobe lesion that underwent postsurgical treatment with Gamma Knife radiosurgery (GKRS) followed by initiation of pembrolizumab five weeks and two days later. From left to right, magnetic resonance images obtained at one month, three months, six months, nine months, and 12 months status post GKRS. Interval increase, decrease, and stability in T2 signal and surrounding enhancement denoted with arrows at bottom of images.

A 50-year-old man with a four-year history of known intracranial metastatic melanoma last resected three years prior to his current presentation, presented to clinic with gait and mobility abnormalities, left visual field deficit, and generalized muscle weakness. An MRI revealed a midline right occipital lesion obscured by a large hemorrhagic lesion in the right cuneus, with surrounding edema and mass effect on the neighboring corpus callosum and right lateral ventricle (Figure 4). The patient subsequently underwent resection, and the pathologic diagnosis was consistent with metastatic melanoma. Postoperative MRI within 24 hours of resection revealed a nonspecific T1-hyperintensity with adjacent enhancement along the posteromedial aspect of the resection cavity. GKRS was then performed to the cavity and pembrolizumab was started five weeks and two days thereafter. As illustrated in Figure 5, MRIs at one month and three months status post GKRS revealed persistent, but stable surrounding enhancement (2.4 x 2.6 x 2.4 centimeters) at the occipital lesion. After six months, imaging revealed a substantial increase in peripheral enhancement surrounding the occipital lesion and increased surrounding parenchymal edema (4.8 x 4.0 x 4.1 centimeters) with new 4 mm leftward midline shift. These findings were noted to be concerning for progression versus radiation-induced necrosis. At nine months (3.9 x 3.5 x 3.0 centimeters) and 12 months status post GKRS, imaging revealed continued decrease in edema and enhancement with no new lesions.

Case 2

**Figure 6:**
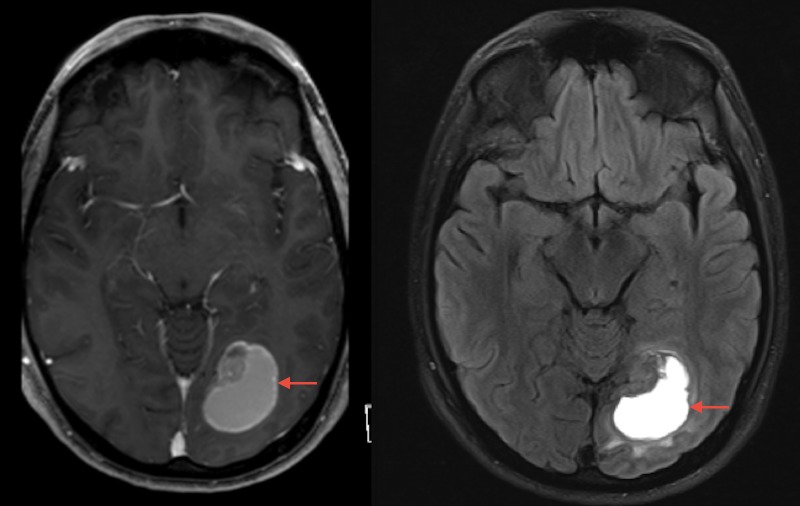
Preoperative magnetic resonance imaging demonstrating post-contrast T1-weighted (left) and T2 fluid attenuated inversion recovery (right) images of 4.3 x 3.1 x 3.4 centimeter enhancing heterogeneous mass lesion in the left occipital lobe.

**Figure 7:**
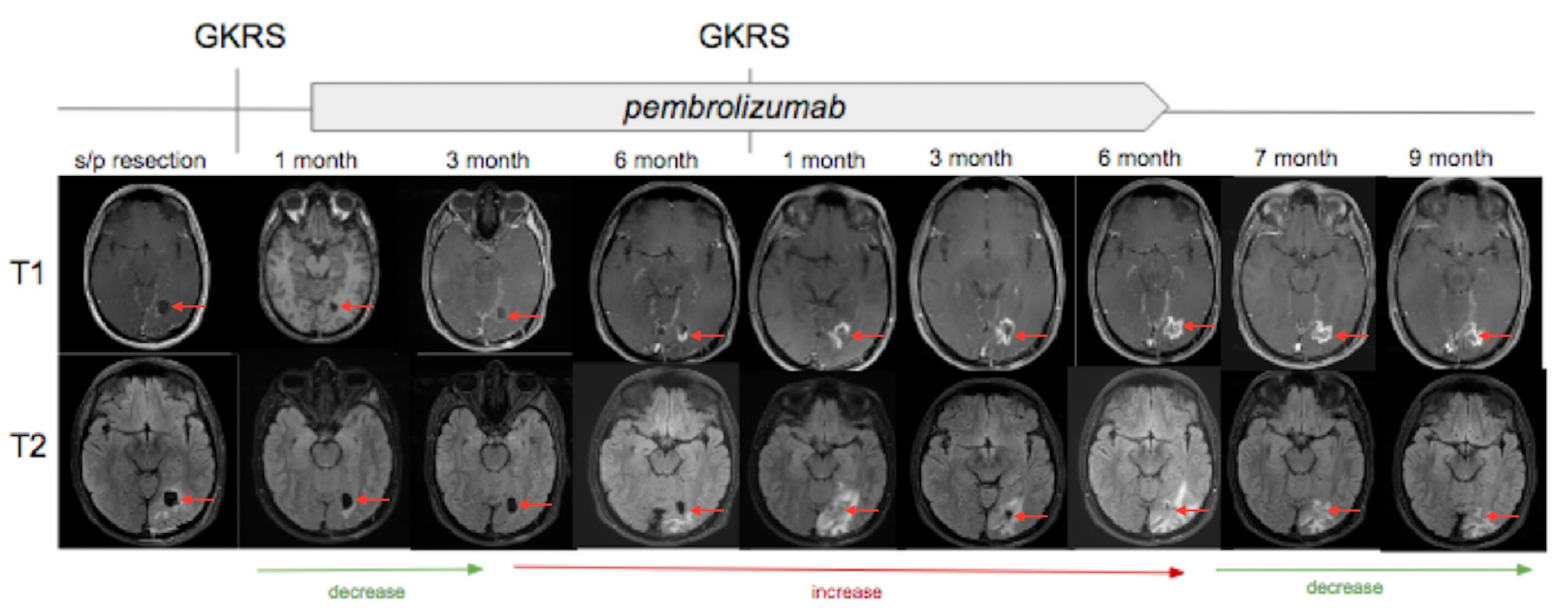
T1-weighted with gadolinium (top) and T2 fluid attenuated inversion recovery images (bottom) associated with the left occipital lobe lesion that underwent postsurgical treatment with Gamma Knife radiosurgery (GKRS) followed by initiation of pembrolizumab one week and two days later. From left to right, magnetic resonance images obtained at one month, three months, and six months status post first GKRS treatment. Repeat-GKRS was then performed while concurrent treatment with pembrolizumab. From left to right, magnetic resonance images obtained at one month, three months, six months, seven months, and nine months status post repeat-GKRS. Interval increase and decrease in T2 signal and surrounding enhancement are denoted with arrows at bottom of images.

A 25-year-old man with a two-year history of melanoma presented with severe headaches, nausea, chills, and left facial paresthesia. An MRI was obtained which revealed an enhancing heterogeneous mass in the left occipital lobe concerning for metastatic melanoma (Figure 6). The patient underwent resection the following day and pathology revealed a metastatic amelanotic melanoma. GKRS was performed less than one month later to the left occipital lesion as well as a left cavernous sinus metastasis, and PD1-antagonist immunotherapy was started nine days later. As illustrated in Figure 7, MRIs at one month and three months after GKRS revealed an interval decrease in the surrounding T2 signal and enhancement. However, new contrast enhancement and associated vasogenic edema was noted on imaging performed at six months.

The aforementioned findings were concerning for tumor progression, and the patient underwent repeat-GKRS to the occipital lesion approximately seven months after the first stereotactic radiosurgery. Imaging at one month status post repeat-GKRS revealed an interval increase in the degree of enhancement and surrounding T2 hyperintensity within the adjacent white matter (2.0 x 2.1 x 3.6 centimeters), with persistent but decreased size of a small focus of internally restricted diffusion as well as increasing internal hemorrhagic components. It was at this time pseudoprogression was first proposed. At three months, his MRI revealed the relatively stable size of the lesion (2.8 x 2.2 x 2.6 centimeters), making pseudoprogression even more likely. Follow-up MRI at six months status post repeat-GKRS revealed an interval increase in the degree of enhancement surrounding the targeted site, but subsequent imaging revealed an overall decrease in mass effect and a decrease in the surrounding T2 hyperintense signal. Over the next five months, concern for pseudoprogression continued to be documented, as sequential imaging revealed regression and eventual stabilization in tumor size and perilesional edema.

## Discussion

Melanoma has a propensity to spread to the brain, even years after a complete resection without evidence of spread. Traditionally, the median survival of patients with brain metastases from melanoma was anywhere from three weeks up to three months in the absence of treatment [14-15]. Through various treatment options including surgery, WBRT, chemotherapy, and GKRS, median survival has improved to three to twelve months [16-17]. The development of even newer targeted therapies, such as PD-1 inhibitors, have recently gained approval for the treatment of metastatic melanoma after surpassing one year in overall median survival in select patient populations [18-20]. Although current data is limited, the combined effects of GKRS and PD-1 inhibitors appear to be even more efficacious to patient longevity.

Since radiosurgery can deliver highly precise irradiation to targets within the brain and inactivate tumor cells biologically, lack of tumor growth is typically considered to be treatment success. However, targeted radiation therapy may also lead to a systemic and vigorous immune response which may affect tissue outside of GKRS-targeted lesions. Better known as the abscopal effect, there is evidence to suggest that targeted radiotherapy in the proper setting can produce a robust immune-mediated effect that may eradicate disease in patients who develop metastatic cancer [21]. If used as an adjuvant to GKRS, immunotherapeutics have the potential to amplify the abscopal effect even further, which is a rationale for their combined use [22]. Currently, interactions between GKRS and the newly-approved PD-1 inhibitors remain largely underinvestigated, but analysis of MRI findings following the use of these therapies may provide a solid foundation for future studies. This analysis includes assessing for disease progression and any findings of pseudoprogression related to PD-1 antagonists or radiation-induced necrosis.

In our retrospective analysis of patients who received both GKRS and anti-PD-1 therapy within fewer than six weeks of each other, 13 subjects had data available for interpretation and nine of those demonstrated progression upon sequential imaging. Of particular importance, two subjects who had imaging findings concerning for progression subsequently had resolution of those changes on follow-up imaging. More specifically, both subjects demonstrated stable or increased enhancement from one month to six months post-radiation with regression in tumor size and/or degree of enhancement that started to occur at some point prior to the nine-month post-GKRS follow-up MRI.

These findings are important because they begin to shed light on the clinical and radiographic effects when patients are subjected to both radiation therapy and PD-1 immunotherapy. As previously mentioned, findings of radiation-induced necrosis from GKRS typically present months to years later on MRI, while immune-mediated responses from PD-1 antagonists more commonly present within three months after administration [23-27]. Since pseudoprogression was noted to occur prior to six months with eventual regression by nine months post GKRS, these findings suggest immunotherapy may accentuate or accelerate radiation-induced changes to surrounding brain tissue. By utilizing two distinct mechanisms against metastatic disease, the cytotoxic effects of radiation and immune-related effects enhanced by PD-1 immunotherapy appear to result in a quick and robust antitumor response.

In terms of clinical response to metastatic melanoma, the use of pembrolizumab or nivolumab alone have demonstrated durable responses, high response rates, and favorable safety profiles in the Keynote and CheckMate clinical trials, respectively. However, there is limited information pertaining to the benefits and harm when administered in combination with GKRS. One study by Liniker, et al. demonstrated no detectable excess toxicity when GKRS was combined with either pembrolizumab or nivolumab [28]. Another study by Ahmed, et al. demonstrated that stereotactic radiation and nivolumab were well-tolerated in the majority of subjects with metastatic melanoma, with the exception that in 11% of patients there was an increase in hemorrhage and/or perilesional edema, defined as ≥20% increase in cerebral metastatic lesion volume [29]. Based on these results, it would be reasonable to assume that simultaneous treatment of multiple brain metastases with GKRS and PD-1 inhibitors could lead to malignant intracranial pressures.

Although the information presented above is limited because pembrolizumab and nivolumab were approved for metastatic melanoma by the Federal Drug Administration (FDA) in mid-2017, one underlying theme of this study focuses on pseudoprogression versus true tumor progression. If researchers and medical personnel are able to differentiate these two phenomena in a more objective manner, oncologists will not be persuaded to stop the use of PD-1 inhibitors because of a presumed treatment failure. In other words, what is assumed to be a treatment-resistant cancer may simply be the way in which an enhanced immune system manifests itself on imaging. Continuing the current treatment regimen would obviously be ideal and ultimately improve survival because there is evidence the immunotherapy is inducing anti-tumor effects. Similarly, if combined treatments repeatedly demonstrate the aforementioned abscopal effect, continued use will likely improve overall survival through systemic disease eradication. Finally, there would be a reduction in potential need for treatment as patients will not need intervention for ‘progressive disease.’ In this manner, accurate and diagnostic identification of pseudoprogression would reduce the inclination to subject the patient to biopsy, eliminating the risks and complications that come with any surgery. Being able to differentiate these findings on imaging alone would obviously provide a clearer treatment plan and eliminate the need for unnecessary interventions, but our current tools make this decision often unclear. Since pseudoprogression is often only confirmed by watchful waiting, and occasionally by biopsy, most of the time we are waiting for regression or stabilization of previously-treated GKRS targets to occur. Better diagnostic methods need to be developed to distinguish these progressions earlier in the treatment of metastatic disease.

Limitations

There were several limitations in our study. Neither of the patients with radiographic pseudoprogression underwent biopsy, thus there was no tissue diagnosis of treatment effect. However, with a biopsy or even resection, there remains a possibility that no metastatic disease remains, as this may be missed on a small biopsy or not completely examined by a pathologist. Consequently, imaging findings may be more reliable after better description of the expected findings are reported.

Although there is no definitive diagnostic test for distinguishing pseudoprogression from tumor progression, diffusion weighted imaging with higher apparent diffusion co-efficient (ADC), magnetic resonance (MR) spectroscopy, and positron emission tomography (PET) may be performed, but their use is controversial due to low sensitivity and specificity. MR perfusion is another imaging technique that has been labeled as an unreliable test due to inconsistent results, but recent evidence suggests otherwise. In April 2017, Umemera, et al. analyzed dynamic contrast-enhanced T1 magnetic resonance imaging (DCE-MRI) in patients being treated with immunotherapy for melanoma brain metastases. In their analysis, pseudoprogression was defined as neurological and radiographic stability/improvement without any new treatment for greater than or equal to two months, and disease progression was confirmed by histopathology. Their results demonstrated a significantly lower median 90th percentile plasma volume (Vp90) on DCE-MRI perfusion studies in pseudoprogression compared to true tumor progression (Umemura Y, Wang D, Peck K, et al., Neuro Oncol. 2017, 19). While monitoring with serial imaging appears to be the most effective strategy thus far, the addition of MR perfusion analysis to subjects in this study may allow for a better predictor of pseudoprogression.

Several patients in this study did not have follow-up MRIs after their GKRS treatment. This was either due to limited MRI access at the institution in which the study was being conducted or related to the loss of follow-up with the included subjects. Since metastatic melanoma is a disease that quickly progresses, many subjects succumbed to their disease before additional imaging could be utilized to assess changes on imaging.

## Conclusions

The combination of GKRS and PD-1 immunotherapy within fewer than six weeks of each other appears to lead to more rapid development of pseudoprogression. This data suggests there is need for further investigation of the role for concurrent treatment with PD-1 inhibitors and GKRS to enhance the treatment of metastatic melanoma. Future studies focusing on the ideal timing for treatment of these two modalities will provide more information regarding their effects.
